# Crosstalk between protein N-glycosylation and lipid metabolism in *Saccharomyces cerevisiae*

**DOI:** 10.1038/s41598-019-51054-7

**Published:** 2019-10-09

**Authors:** Antonisamy William James, Chidambaram Ravi, Malathi Srinivasan, Vasanthi Nachiappan

**Affiliations:** 1 Biomembrane Lab, Department of Biochemistry, School of Life Sciences, Bharathidasan University, Tiruchirappalli, 620 024 Tamilnadu India; 2 Department of Lipid Science, CSIR-Central Food Technological Research Institute (CSIR-CFTRI), Mysore, 570020 India

**Keywords:** Biochemistry, Glycobiology

## Abstract

The endoplasmic reticulum (ER) is a multi functional organelle and plays a crucial role in protein folding and lipid biosynthesis. The *SEC59* gene encodes dolichol kinase, required for protein glycosylation in the ER. The mutation of *sec59-1* caused a protein N-glycosylation defect mediated ER stress resulting in increased levels of phospholipid, neutral lipid and sterol, whereas growth was reduced. In the *sec59-1∆* cell, the N-glycosylation of vacuolar carboxy peptidase-Y (CPY) was significantly reduced; whereas the ER stress marker Kar2p and unfolded protein response (UPR) were significantly increased. Increased levels of Triacylglycerol (TAG), sterol ester (SE), and lipid droplets (LD) could be attributed to up-regulation of *DPP1*, *LRO1*, and *ARE2* in the *sec 59-1∆* cell. Also, the diacylglycerol (DAG), sterol (STE), and free fatty acids (FFA) levels were significantly increased, whereas the genes involved in peroxisome biogenesis and Pex3-EGFP levels were reduced when compared to the wild-type. The microarray data also revealed increased expression of genes involved in phospholipid, TAG, fatty acid, sterol synthesis, and phospholipid transport resulting in dysregulation of lipid homeostasis in the *sec59-1∆* cell. We conclude that *SEC59* dependent N-glycosylation is required for lipid homeostasis, peroxisome biogenesis, and ER protein quality control.

## Introduction

Proteins synthesized in the ER are post-translationally modified by N-glycosylation and O-glycosylation. The glycosylation process starts in the ER, and extends to the Golgi apparatus, which requires activated dolichol precursors for the initial steps. The vast majorities of secretory proteins are N-linked glycoproteins and play an important role in protein secretion, morphogenesis, and development of multi-cellular organisms. *SEC59* (SECretory) encodes dolichol kinase (DK) and catalyzes CTP-dependent phosphorylation of dolichol. Dolichol kinase transfers the phosphoryl group from CTP to dolichol and catalyzes the final step of the *de novo* pathway for the Dol-P formation. CTP-mediated dolichol kinase is involved in recycling of glycosyl carrier lipid after it is discharged as Dol-P-P in N-glycosylation reactions. On the ER luminal surface the Dol-P-P phosphatase converts Dol-P-P to Dol-P, and is diffused back to the cytoplasmic leaflet of the ER. Dol-P (Dolichol Monophosphate) serves as a glycosyl carrier lipid in the assembly of N-linked glycoproteins, glycosylphosphatidylinositol anchors, and C and O-mannosylation. The DK is involved in the dolichol monophosphate (Dol-P) synthesis and serves as a carrier lipid in the assembly of N-linked glycoproteins^1^. When the accumulation of unfolded or misfolded proteins in the ER activates the transmembrane kinase/nuclease Ire1p^2^, and initiates the *HAC1* mRNA splicing. The Hac1p, a βZIP transcription factor induced the unfolded protein response (UPR) target genes^2^. The activation of the UPR allows the cell to tolerate stress and presumably assist in a correction of the insult that is caused by unfolded protein accumulation^2^. During the ER stress, the misfolded or unfolded proteins are accumulated in the ER, further transported to the cytosol, and eliminated by the proteasomal degradation^3^. When there is a defect in protein folding, the ER-associated degradation (ERAD) is enhanced. The UPR and ERAD pathways are responsible for removal of the aberrant proteins from the ER^3^. The protein glycosylation and protein quality control homeostasis are crucial and evolutionarily conserved from yeast to human^4^. The N-glycosylation defects are associated with human metabolic diseases, congenital disorder of glycosylation (CDG), muscular hypotonia and progressive dilative cardiomyopathy^5^. The major proportions of lipids are synthesized in the ER and transports to their destiny for various organelles function. The storage lipids TAG and cholesterol esters are associated with atherosclerosis and liver steatosis in human. The impact of the protein glycosylation defect on lipid metabolism (the phospholipid, neutral lipid, sterol, and fatty acid metabolism), and its associated human lipid metabolic disorder remains elusive. A genome-wide screening study for a protein glycosylation defect in *S. cerevisiae* may provide a novel idea for identification of glycosylation defect linked lipid disorders. The systematic interrogations of cellular pathways will help to identify the crosstalk between protein glycosylation and lipid metabolism.

## Results

### *SEC59* is involved in cell growth and membrane morphology

**Figure 1:**
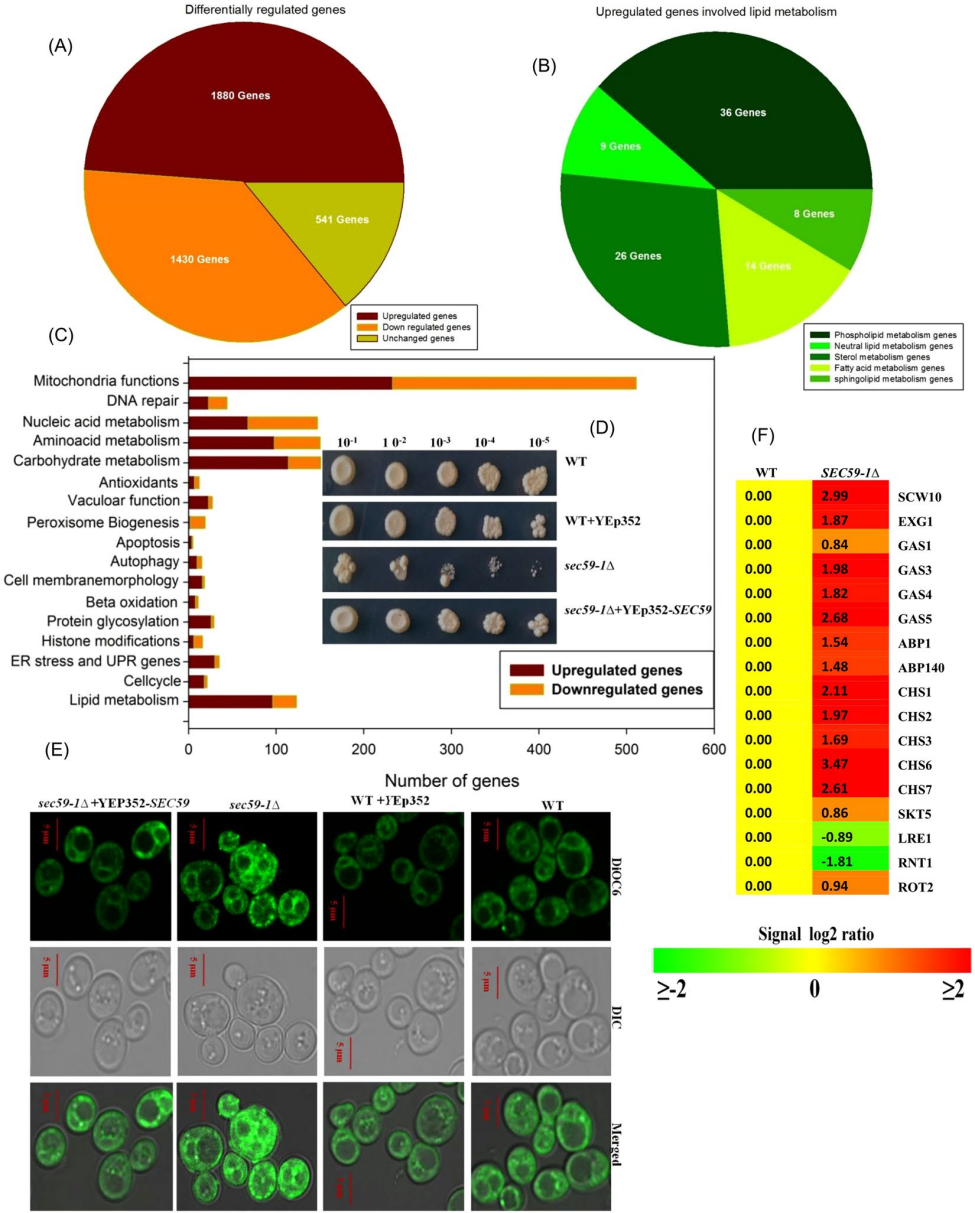
The Sec59p required for growth and membrane morphology. (**A**) Microarray analysis of differentially regulated genes in *sec59-1∆* cell. (**B**) Distribution of differentially regulated genes involved in lipid metabolism in *sec59-1∆* cell. (**C**) Differentially regulated genes involved in various functions in *sec59-1∆* cell. The microarray gene expression data were analyzed and expressed in fold change expression values are provided as log base 2. From the log base values greater than 0.6 fold is statistically significant (*p < 0.05). (**D**) Wild-type, wild + YEp352, *sec59-1∆* and *sec59-1∆* + YEp352-*SEC59* cells were grown in SC or SC-U medium at 30 °C until mid-log phase. Equal amount of cells was serially diluted and three μl of cells were spotted on SC or SC-U agar plates and incubated for 48 h at 30 °C and the growth pattern was observed. (S1 A) For growth curve analysis, cell growth was studied by measuring the A_600 nm_ of cells at frequent time intervals until 24 h. (S1B) We have analyzed 50 fields for each sample, the cells with membrane defects were counted, and the values are expressed per 100 cells. (**E**) Membrane staining. Wild-type, *sec59-1∆* and *sec59-1∆* + YEp352-*SEC59* cells were grown in SC or SC-U medium at 30 °C up to mid-log phase, and the cell membrane stained using the lipophilic dye DiOC6, as described in the methods section. Fluorescence imaging was performed on confocal microscope (LSM 710-Zeiss) equipped with a 100/1.40 oil objective and an AxioCam MRM camera (Zeiss). Bar, 5 μm. (**F**) Microarray expression of genes involved in the cell wall synthesis and assembly.

The microarray result shows that 1880 genes involved in various functions were upregulated and 1430 genes were down regulated in the *sec59-1∆* strain (S1). Among these, 92 genes involved in lipid metabolism were upregulated (Fig. 1A–C). The *sec59-1Δ* cell growth was severely affected even at the permissible temperature until 48 h, and the growth phenotype was rescued by the overexpression of YEp352-*SEC59* (Figs 1D and S1). Hence the membrane morphology was examined in the *sec59-1Δ* cell using the lipophilic fluorescent dye DiOC6 (3,3-dihexyloxacarbocyanine iodide) that stains the plasma membrane, ER and Golgi vesicles^6^. The ER and vacuolar membrane were highly vesiculated and formed clumped aggregates (Fig. 1E), and the overexpression of YEp352-*SEC59* in the *sec59-1∆* cell restored the fragmented vacuolar structure, clumped aggregation of membranes and fragmented membrane punctate-like structure (Fig. 1E). The microarray result shows the genes involved in cell wall synthesis and assembly (*SCW10, EXG1, GAS1, GAS3, GAS4, GAS5, CHS1, CHS2, CHS3, EXG1, SCS3, CHS6*, and *CHS7*) were up-regulated in the *sec59-1Δ* cell (Fig. 1F) revealing that Sec59 plays a vital role in cell growth and membrane maintenance in *S. cerevisiae*.

### The UPR and ER stress induced in the *sec59*-1∆ cell

**Figure 2:**
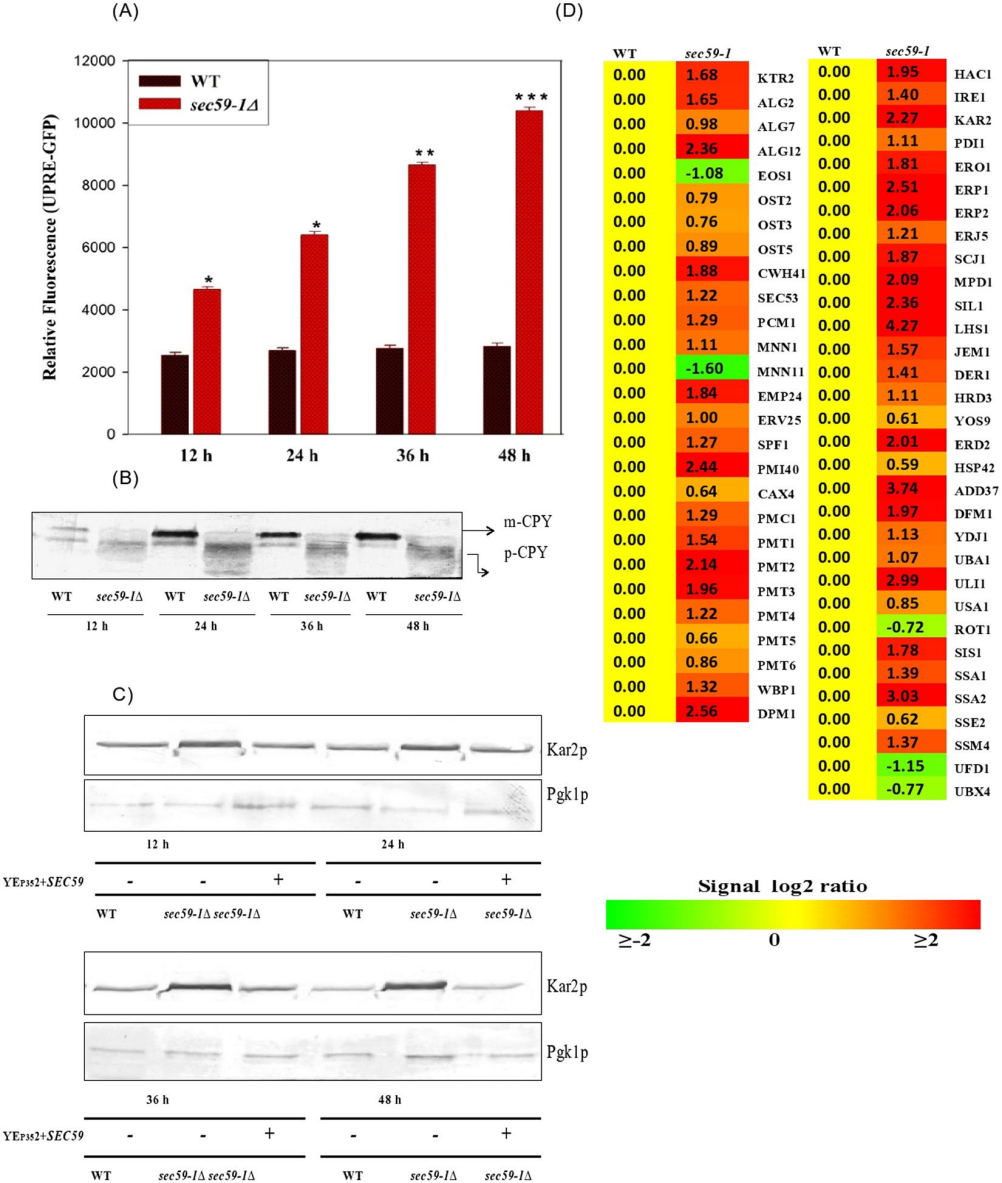
Measurement of UPR in wild and *sec59-1∆* cells. (**A**) The vector pRS314 bearing UPRE-GFP was expressed in the wild-type and *sec59-1*∆ cells, and grown at 30 °C in synthetic complete media devoid of tryptophan (SC-Trp). Equal amount of cells were collected every 12 h to 48 h and the activated UPRE-GFP was measured using F-4500 fluorescence spectro-photometer (excitation at 488 nm and emission at 510 nm). The wild-type control cells were compared with the *sec59-1*∆ cells and the data represents the mean ± SD (*P < 0.05, **P < 0.01 and ***P < 0.001) of triplicates from three separate experiments. (B) Glycosylation status of carboxypeptidase-Y. Wild-type and *sec59-1∆* cells were grown in YPD medium at 30 °C. The cells were collected every 12 h, lysed and an equal amount of protein (50 μg) was separated by 8% SDS page as described in detail in the methods section. Anti-CPY was used to check the glycosylation status of CPY expression in a time-dependent manner. (**C**) The expression of Kar2 protein. The wild-type, wild + YEp352, *sec59-1∆* and *sec591-1∆* + YEp352-*SEC59* cells were grown in SC or SC-U medium until mid-log phase at 30 °C. The cells were lysed and equal amount of protein (50 μg) were separated by 8% SDS page as described in the methods section. Anti–Kar2 antibody was used to analysis the Kar2p expression and anti-Pgk1 was used as a loading control. **(**S1C) The original Western blot picture was scanned by Bio-Rad gel doc with a black-and-white gel tray, and the protein band was quantified with Image-J software. Kar2p expression was normalized with Pgk1p, and the data represent the means ± SD (*P < 0.05) of triplicates from three separate experiments (**D**) Microarray expression of genes involved in protein glycosylation, protein folding, and ER stress marker genes.

The *sec59-1* mutant cell shows severe N-glycosylation defect in the vacuolar carboxy peptidase-Y (CPY) protein from mid-log phase (12 h) to stationary phase (48 h) (Fig. 2B). The UPR was also significantly increased until 48 h as measured by the expression of the UPRE-GFP fluorescence (Fig. 2A).

The expression of ER chaperone, Kar2p was increased in the *sec59-1∆* cell until 48 h, and the overexpression of YEp352*-SEC59* restored the ER stress (Fig. 2C). Also, the microarray result shows that 14 genes (*HAC1, IRE1, KAR2, PDI1, ERO1, SCJ1, MPD1, SIL1, LHS1, YOS9, HSP42, ERJ5, ERP1, ERP24*, and *ERV25*) involved in protein folding, ERAD pathway, and ER chaperone were upregulated (Fig. 2D). Among these genes, the *LHS1* gene expression was up-regulated by ∼4.27 fold and *HAC1, KAR2, SIL1, ERP1*, and *ERP2* genes were elevated by more than ∼2.0 fold, and rest of the genes (*IRE1, PDI1, ERO1, SCJ1, MPD1, HRD1, YOS9, DER1, HSP42, ERJ5, EMP24*, and *ERV25*) were up-regulated by ∼1.5 fold. The *EMP24* and *ERV25* expression were increased by ∼1.84 and ∼1.0 fold (Fig. 2D). The expressions of ERAD pathway genes (*DOA10, DFM1, RSP5, UBX4, UFD1, HRD3, USA1, DER1*, and *ADD37*) were significantly upregulated thereby eliminating the misfolded or unfolded proteins in the *sec59-1∆* cell (Fig. 2D). The N-glycosylation defect observed in the *sec59-1∆* cell was sustained until 48 h (Fig. 2B) despite the genes involved in protein glycosylation (*EOS1, WTM1, KTR2, ROT2, ALG2, OST2, OST3, OST5, ALG7, PCM1, PMT1, PMT2, PMT3, PMT4, PMT6, CWH41, ALG12, WBP1, BST1*, and *DPM1*) being significantly upregulated (Fig. 2D). From this result, we can depict that the *Sec5*9 plays a crucial role in protein glycosylation and ER protein quality control.

### Role of *SEC59* in glycerophospholipid metabolism

**Figure 3:**
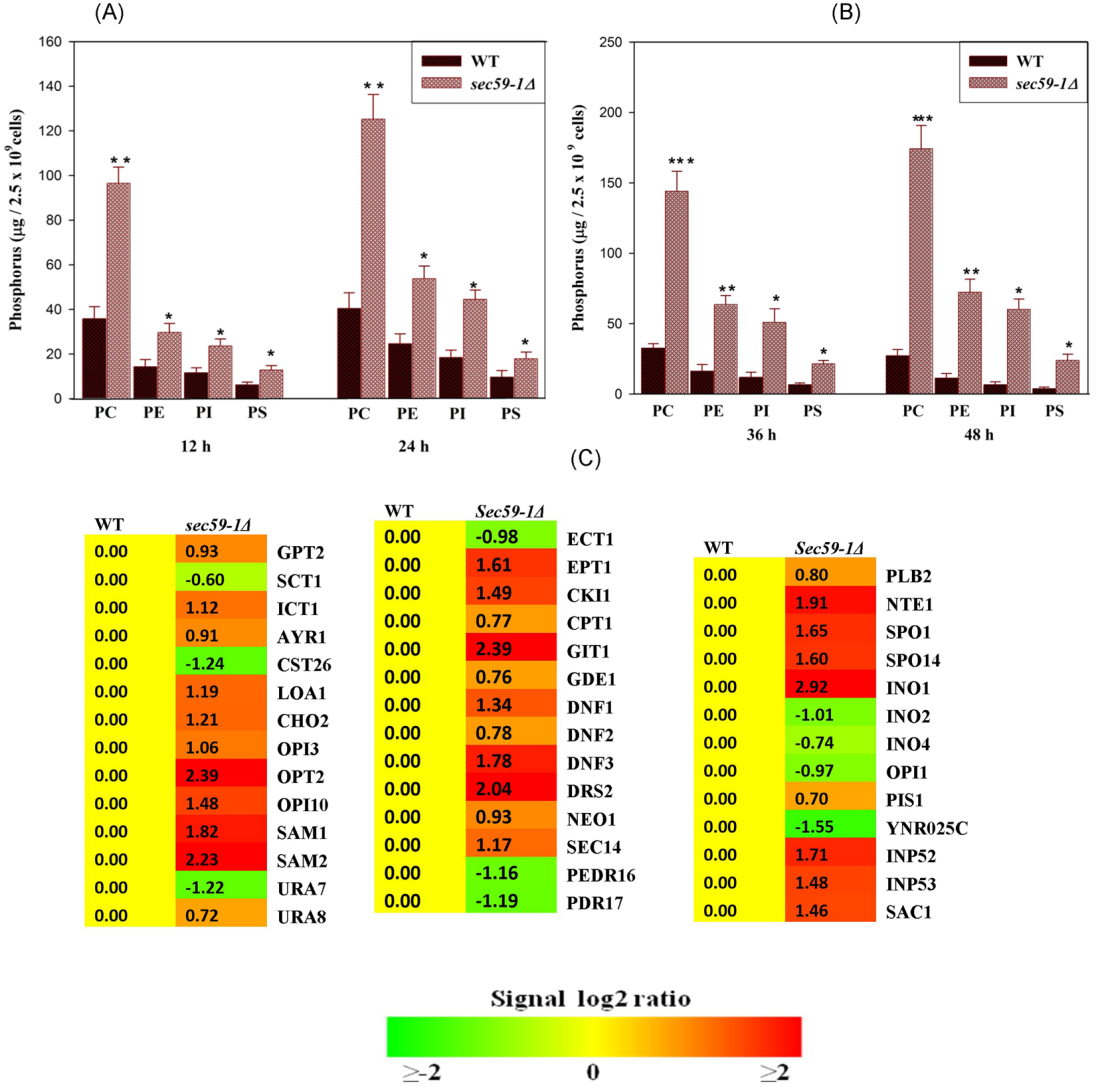
Time-dependent study of phospholipids. (**A**,**B**) The wild-type and *sec59-1*∆ cells were grown  in YPD medium at 30 °C. Equal amount of cells were harvested in a time dependent manner (12 h to 48 h), lipids were extracted and the individual phospholipids separated by two-dimensional TLC and the phosphorus was quantified as described in the methods section. (**C**) Expression of genes involved in phospholipid metabolism. The wild-type cells at different time intervals were compared with their respective *sec59-1*∆ cell and the data represents the mean ± SD (*P < 0.05, **P < 0.01 and ***P < 0.001) of triplicates from three separate experiments.

The *SEC59-1* mutation causes the accumulation of misfolded or unfolded proteins induced ER stress. Also, the membrane lipids and storage lipids are majorly synthesized in the ER. Hence we sought to identify the impact of Sec59p defect in lipid metabolism. The phospholipids, phosphatidyl choline (PC), phosphatidyl ethanolamine (PE), phosphatidyl inositol (PI), phosphatidyl serine (PS) and minor phospholipids, lysophosphatidylcholine (LPC), phosphatidic acid (PA), and cardiolipin (CL) levels were significantly increased in *sec59-1Δ* cell compared to the wild-type (Fig. 3A). The time lapse study also shows that, PC, and PE were increased at 36 h and 48 h (Fig. 3B), also, minor phospholipids were significantly increased in the *sec59-1∆* cell until 48 h, whereas they were reduced in the wild-type cell.

Choline is a precursor for phosphocholine synthesis^7,8^. The microarray data revealed that genes involved in PC, and PE synthesis (*CHO2, OPI3, CPT1, EPT*, and *CKI1*) were up-regulated, and rest of the genes (*CDS1, CHO1, PSD1, PSD2, PCT1*, and *EKI1)* were not significantly altered in *sec59-1Δ* cell (Fig. 3C). However, the *SAM1* and *SAM2* (S-adenosylmethionine synthetase) were up-regulated by ∼2.0 fold in *sec59-1Δ* cell, which acts as a methyl donor, and is required for the PC synthesis by the activation of Cho2p and Opi3p. The *URA7* gene was down regulated by 1.22 fold, whereas *URA8* (CTP synthase) was up-regulated in *sec591-Δ* cell, which are required for the synthesis of PC through the activation of the phosphocholine cytidyltransferase in CDP-choline pathway^9,10^. The phospholipid precursors (LPA and PA) are required for synthesis of the phospholipid through CDP-DAG pathway. The genes involved in LPA and PA synthesis (*GPT2, AYR1, LOA1*, and *ICT1*) were up-regulated (Fig. 3D); and the phospholipid levels were increased in the *sec59-1∆* cell until 48 h even though the phospholipases (*SPO14, NTE1*, and *PLB2*) were up-regulated.

The lipid transporters and aminophospholipid flippase of the plasma membrane provide the substrate for phospholipid synthesis from exogenous supplementation. The expression of plasma membrane permeases (*GIT1, DNF1, DNF2*, and *DNF3*), and aminophospholipid flippase (*NEO1, SEC14, PDR16, PDR17*, and *DRS2)* were increased in the *sec59-1Δ* cell. The *GIT1* encodes a permease, which is involved in uptake of glycerophosphoinositol and glycerophosphocholine^11,12^, and its expression was up-regulated ∼2.5 fold in our study. The *GDE1* (glycerophosphocholine phosphodiesterase) hydrolyzes the GPC to form choline and glycerol phosphate and its expression was up-regulated. The *DNF1* and *DNF2* involved in the transport of PC, PS, and PE from extracellular to maintain the amino-phospholipid asymmetry and their expression was significantly increased in the *sec591-Δ* cell (Fig. 3C). The *SEC14* involved in the transport and regulation of PC, and PI homeostasis and its expression was also up-regulated. The expression of *NEO1* (putative aminophospholipid translocase) was significantly up-regulated and is known to be involved in the phospholipid asymmetry of plasma membrane.

The inositol, inositol phosphate, and inositol-containing phospholipids are signaling molecules required for the synthesis of phospholipid and complex sphingolipids. In the *sec59-1∆* cell, the *INO1* (Inositol 3-phosphate synthase) gene was up-regulated, whereas the *OPI1* (Negative regulator of phospholipid synthesis) was significantly down regulated. The *PIS1* (phosphatidyl inositol synthase) gene was up-regulated, and the expression of phosphatidylinositol phosphate phosphatases (*INP52, INP53*, and *SAC1)* were significantly increased, whereas the *CST26* gene (LPI acyltransferase) was down regulated. The expression levels of *INP52* and *INP53* were higher than *PIS1* in the *sec59-1∆* cell (Fig. 3C). These results suggest that *SEC59* mediated glycosylation is required for phospholipid homeostasis and turnover in *S. cerevisiae*.

### SEC59 plays a crucial role in sterol metabolism

**Figure 4:**
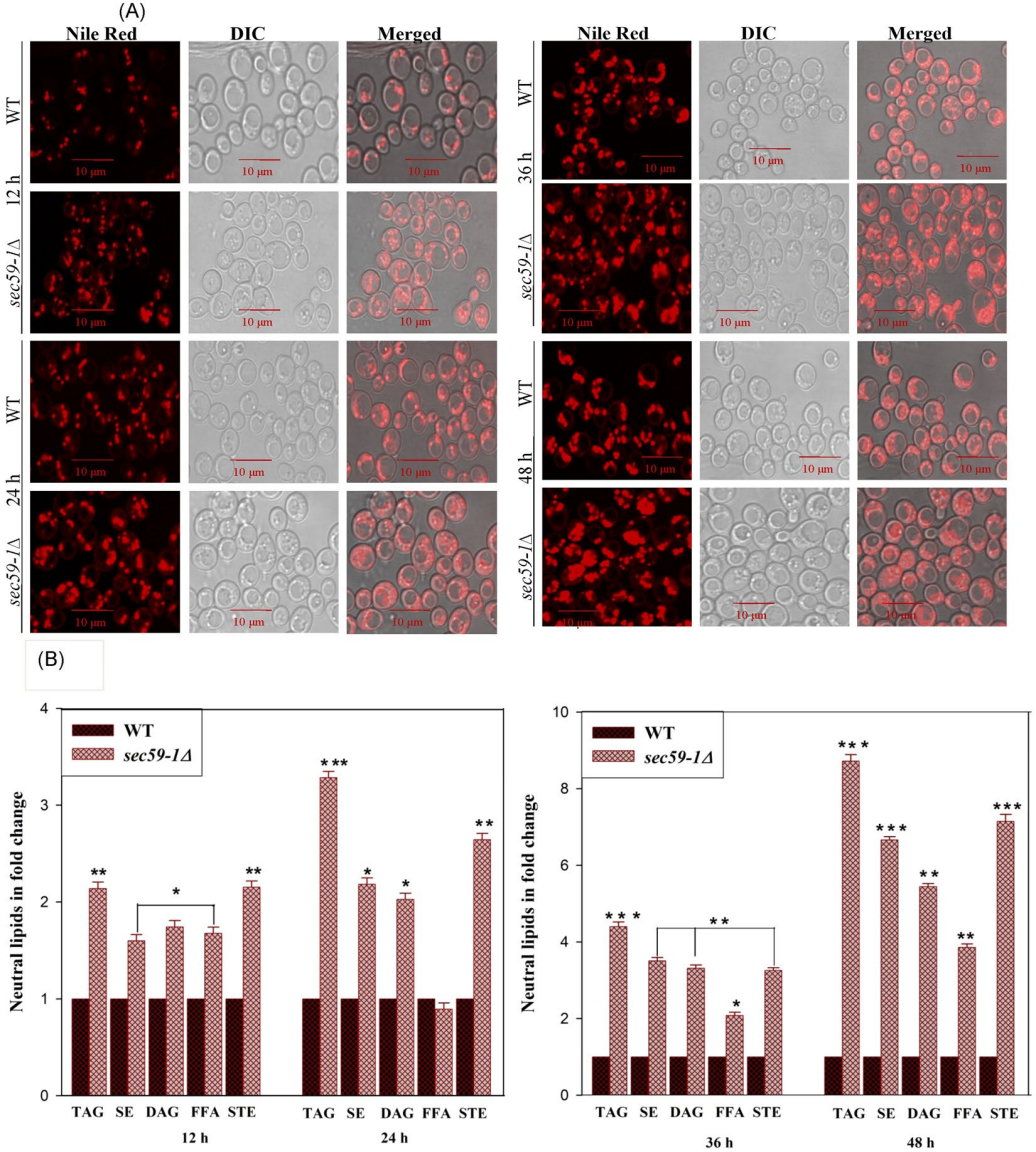
Time dependent LD staining in wild-type and *sec59-1∆* cells. (**A**) The wild-type and *sec59-1*∆ cells were grown in YPD medium at 30 °C. Cells were collected in every 12 h intervals and stained with Nile Red as described in the methods section. Fluorescence imaging was performed on a confocal microscope (LSM 710-Zeiss) equipped with a 100×/1.40 oil objective and an AxioCam MRM camera (Zeiss). Bar, 10 μm. (**B**) Time-dependent study of the neutral lipids. The wild-type and *sec59-1*∆ cells were grown in YPD medium at 30 °C. Equal amounts of cells were harvested in a time-dependent manner (12 h to 48 h), lipids were extracted and the individual lipids were separated by TLC and quantified by densitometry scanning as described in the methods section. The wild-type cells at different time intervals were compared with their respective *sec59-1*∆ cell and the data represents the mean ± SD (*P < 0.05, **P < 0.01, and ***P < 0.001) of triplicates from three separate experiments.

**Figure 5:**
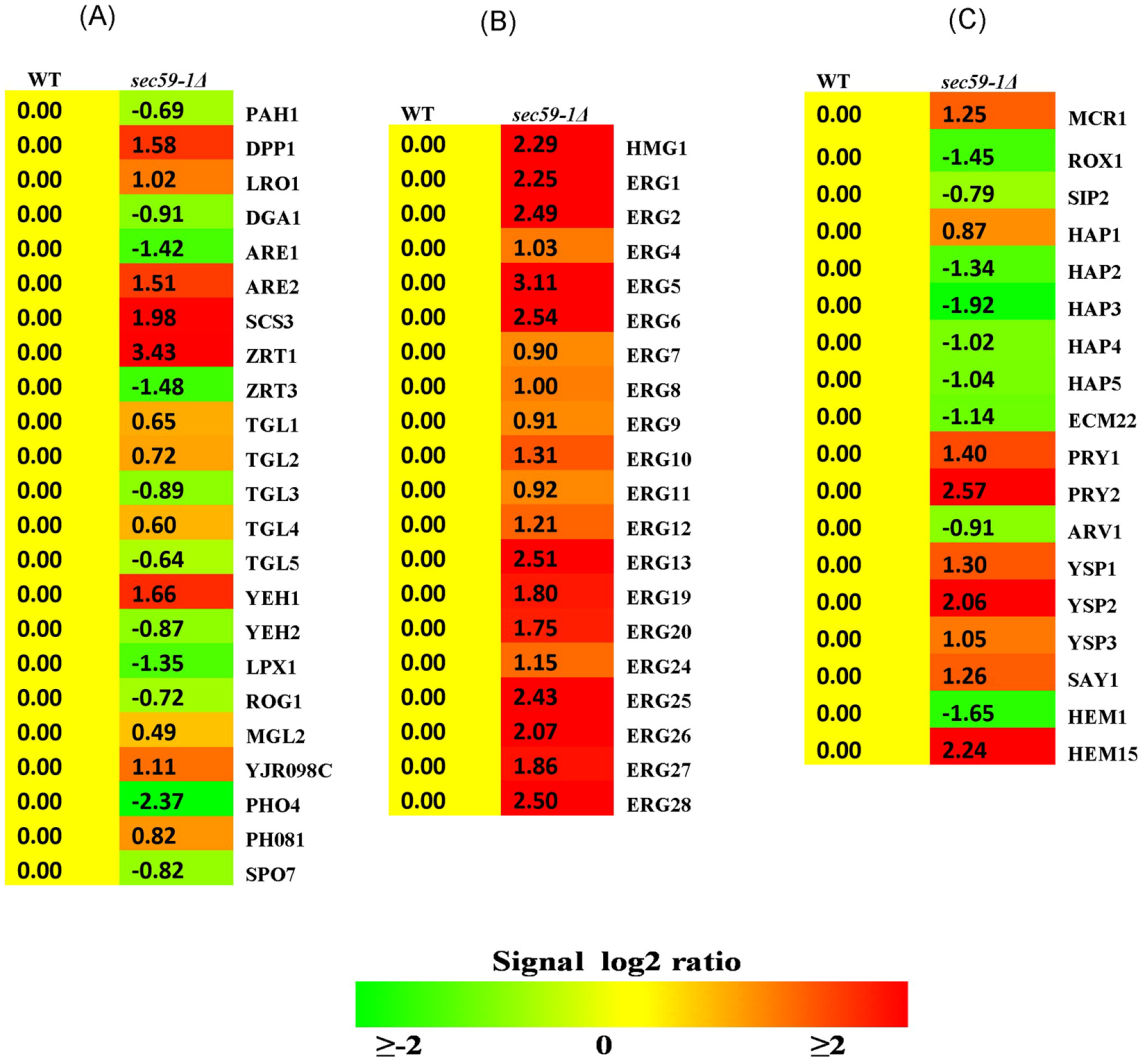
(**A**) Microarray expression of genes involved in the neutral lipid metabolism and sterol metabolism. The microarray gene expression data were analyzed and expressed in fold change expression values are provided as log base 2. From the fold changes values greater than 0.6 fold is statistically significant (*p < 0.05).

The endoplasmic reticulum is a main site for sterol synthesis. Sterol homeostasis is regulated by ER-localized sensors at the transcriptional and post-transcriptional level^13^. Sterol is essential for the viability of eukaryotic cells and membrane integrity but the accumulation of sterol is detrimental to membrane function^14^. Sterol is synthesized through a complicated pathway involves about ∼30 Erg proteins. In the *sec59-1*∆ cell, sterol levels were increased by ∼2.0 fold, ∼4.0 fold and ∼8.0 fold at 24, 36 and 48 h, respectively (Fig. 4B). From the sterol metabolism, 23-genes involved in sterol synthesis were upregulated (*HMG1, ERG1, ERG2, ERG5, ERG6, ERG9, ERG11*, *ERG13, ERG25,ERG28, ERG20, ERG26, ERG27, ERG28, MDV1, ERG4, ERG7, ERG8, ERG9, ERG10, ERG11, ERG12, MCR1*, and *ERG24*) in *sec59-1∆* strain when compared with the wild-type (Fig. 5B). The sterol regulatory element binding proteins (Upc2p and Ecm22p) regulate the sterol biosynthetic genes (*ERG2, ERG3*) and *SIP2* is a negative regulator of sterol synthesis. In the *sec59-1∆* cell, the *ERG2* gene expression was up-regulated, whereas the *ECM22* and *SIP2* were down regulated.

In *S. cerevisiae*, six proteins with the StART (steroidogenic acute regulatory protein-related lipid transfer) like domain (*YSP1, YSP2, SIP3, LAM4, LAM5*, and *LAM6)* are involved in transport of sterol from plasma membrane to ER and conserved in eukaryotes^15^. The expression of *YSP1, YSP2*, and *SIP3* were elevated; whereas the *LAM4, LAM5*, and *LAM6* were not altered in the *sec59-1*∆ cell (Fig. 5C). The accumulation of sterol in the ER induces ER stress, influences protein misfolding, and activates sterol acetylation. The *PRY1*, *PRY2*, and *SAY1* were involved in the export of the acetylated sterol and were up-regulated.

**Figure 6:**
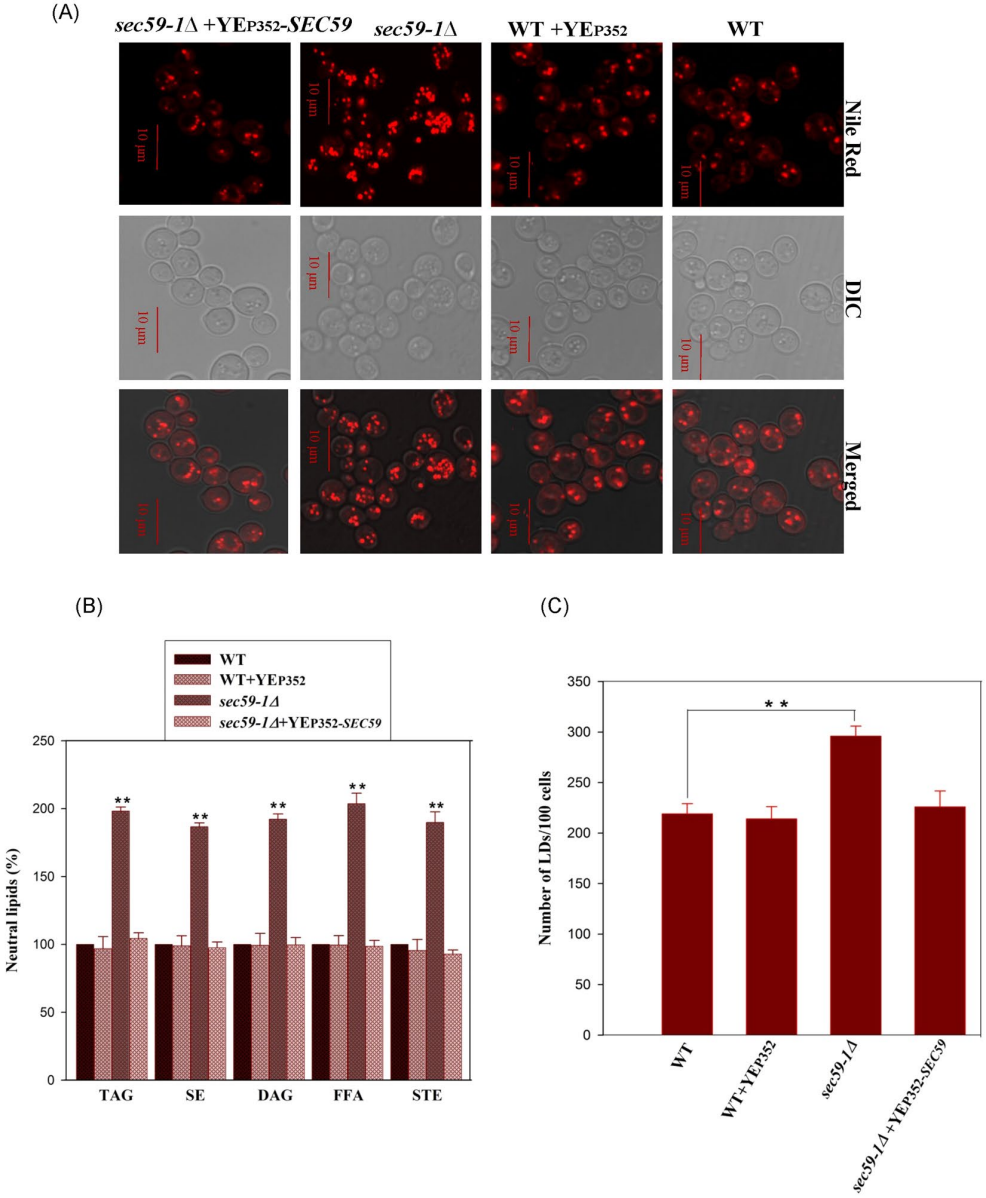
Overexpression of YEp352-*SEC59* in *sec59-1*∆ cell rescues the neutral lipids and LD numbers. (**A**) The wild-type, wild + YEp352, and *sec59-1*∆ + YEp352-*SEC59* cells were grown in SC or SC-U medium at 30 °C up to mid-log phase, and stained with Nile Red as described in the methods section. Fluorescence imaging was performed on confocal microscope (LSM 710-Zeiss) equipped with a 100×/1.40 oil objective and an AxioCam MRM camera (Zeiss). Bar, 10 μm. (**B**) The wild-type, wild + YEp352, and *sec59-1*∆ + YEp352-*SEC59* cells were grown in SC or SC- U medium at 30 °C. Equal amount of cells were harvested in mid-log phase, lipids were extracted and the individual lipids were separated by TLC and quantified by densitometry scanning as described in the methods section. (**C**) We analyzed 50 fields for each sample and counted the LD numbers, and the values are expressed per 100 cells. The wild-type cell lipids were compared to *sec59-1*∆ cell and the data represents the mean ± SD (*P < 0.05, **P < 0.01, and ***P < 0.001) of triplicates from three separate experiments.

Heme plays a central role in the transcriptional regulation of the genes involved in sterol homeostasis^16,17^, and are also required for enzyme activity of Erg3p (C-5 sterol desaturase), and Erg5p^18^. The expression of *HEM1* and its transcription regulators (*HAP2* and *HAP3*) were significantly reduced, whereas *HEM15* (ferrochelatase) was elevated in the *sec59-1∆* cell (Fig. 5C). The 5-aminolevulinate synthase (*HEM1)* is involved in heme biosynthesis and is homologous to human ALAS2, and its mutation causes X-linked sideroblastic anemia. The transcription factor *HAP1* was up-regulated, whereas the *ROX1* (oxygen responsive repressor) was down regulated (Fig. 5C). The transcription factor Hap1p positively regulates the *ERG11* and negatively regulates the Rox1p. The *HMG1* (HMG-CoA reductase) expression was significantly increased, and *HMG2* was not altered in the *sec59-1∆* cell. Finally, the over-expression of YEp352-*SEC59* in *sec59-1∆* strain restored the sterol levels as in the wild-type (Fig. 6B). These results suggest that *SEC59* is involved in the sterol homeostasis.

### Defect in *SEC59* disrupts storage lipid homeostasis

The non-polar lipids TAG and SE are the energy reservoirs, and are stored as LD, which is surrounded by the phospholipid monolayer. The TAG is also required for membrane lipid synthesis. In the *sec59-1* deletion strain, TAG (183%) and SE (178%) were increased, and subsequently, 1, 2-DAG, 1, 3-DAG, and free fatty acids (FFA) were also accumulated (Fig. 4B). The LD number and size were also significantly increased when compared to wild-type. The time-lapse study also showed that TAG, SE, and the LD number drastically increased in the *sec59-1∆* strain (Fig. 4A,B), and subsequently, DAG, free fatty acids and sterol levels were also massively elevated when compared to wild type (Fig. 4B). The overexpression of YEp352-*SEC59* in the *sec59-1∆* cell restored the neutral lipids (TAG, SE, DAG, and STE), LDs, and FFA as in wild-type (Fig. 6A–C). These results strongly reveal that Sec59p is involved indirectly in neutral lipid homeostasis and LD maintenance. The major LD component TAG is synthesized by diacylglycerol acyltransferase (*DGA1*) and lecithin cholesterol acyltransferase [*LCAT/LRO1*]^19,20^. In the *sec59-1∆* cell, the *DGA1* expression was significantly reduced whereas *LRO1* and *YJR098C* expression were elevated. *YJR098C* has lecithin cholesterol acyltransferase (LCAT) motif (Accession ID: PF02450) but with an uncharacterized *ORF* and also could account for TAG accumulation in the *sec59-1∆* cell (Fig. 5A). The TAG and lipid droplets are also involved in synthesis of membrane lipids and act as an energy source during starvation, and are hydrolyzed by TAG lipases. The major TAG lipases, *TGL3, TGL5*, and *LPX1* (peroxisomal TAG lipase) were down regulated whereas as the minor lipases (*TGL2* and *TGL4*) were up-regulated in the *sec59-1∆* cell when compared to the wild-type (Fig. 5A). Another major lipid droplet component is SE, which is synthesized by sterol esterification through the enzyme acyl-CoA: sterol acyltransferases (Are1p & Are2p)^21^. The *ARE1* expression was down regulated, whereas *ARE2* expression was up-regulated when compared to the wild-type. The SE is mainly synthesized through the Are2p (oxygen-dependent sterol esterification), and is hydrolyzed by *YEH1* and *YEH2*^22^. In the *sec59-1∆* cell, the *YEH1* gene was down regulated whereas the *TGL1* and *YEH2* were up-regulated (Fig. 5A). However, the major MAG lipase *YJU3* was not altered but there was repression of MAG lipase *ROG1* whereas the expression of *MGL2* (MAG lipase) was slightly increased in the *sec59-1Δ* compared to the wild-type. These results strongly suggest that, dolichol kinase dependent protein glycosylation defect dysregulates non-polar lipid metabolism.

### SEC59 mutation affects lipid phosphatases and their gene regulation

In *S. cerevisiae*, five lipid phosphatases (*PAH1, DPP1, LPP1, APP1*, and *PHM8)* are involved in the DAG synthesis. The *PAH1* encodes a major Mg^2+^ dependent phosphatidate phosphatase (PA), which dephosphorylates PA to yield DAG and is involved in *de-novo* synthesis of TAG, and LD^23–25^. In the *sec59-1∆* cell, the expression of *DPP1* was significantly increased, and *PHM8* was slightly increased whereas the *PAH1* expression was significantly reduced, and there were no changes observed in *LPP1* and *APP1* expression. The *ZRT1* (Zinc transporter) was up-regulated, whereas the *ZRT3* was down regulated, which led to defect in the transport of zinc from the vacuole to cytoplasm (Fig. 5A). The *PHO4* (transcription factor of phm8) was repressed, but the *PHM8* expression was slightly increased in the *sec59-1* deletion strain when compared to the wild-type. The zinc dependent vacuolar PA phosphatase (*DPP1*) and magnesium dependent LPA phosphatase (Phm8p) provides a substrate for the TAG formation in the *sec59-1∆* cell.

### Effect of protein glycosylation defect on fatty acids of membrane and storage lipids

In the *sec59-1∆* cell, both phospholipids and neutral lipids were increased. In order to investigate the influence of protein glycosylation defect on fatty acids, we subjected the individual phospholipids (PC, PE, PI, and PS) and neutral lipids (DAG, TAG, and SE) to Gas chromatography/Mass spectrometry (GC/MS) analysis (Fig. 6B).

**Figure 7:**
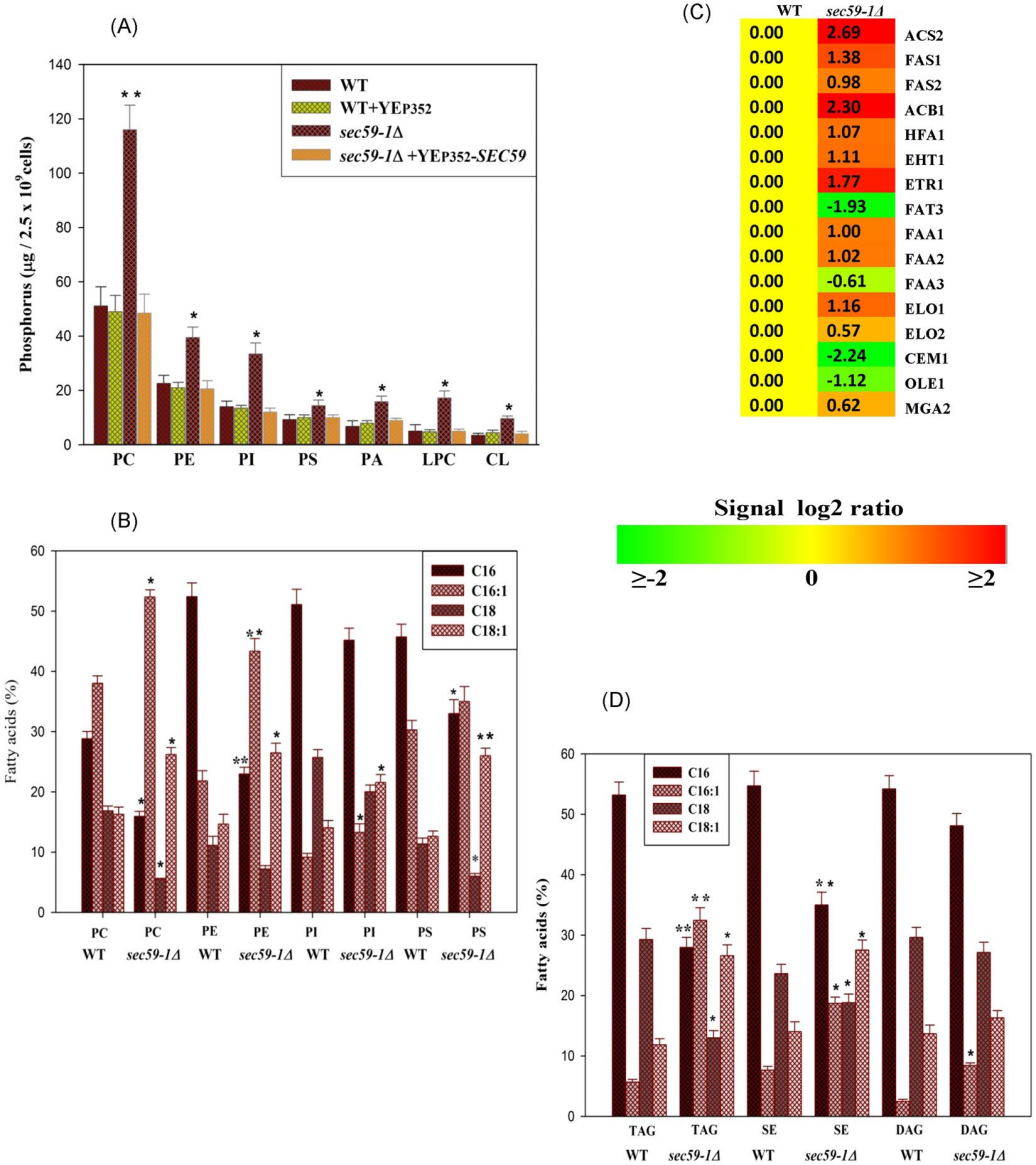
Quantification of phospholipids in wild-type and YEp352-*SEC59* overexpressed cells. (**A**) Wild-type, wild + YEp352, *sec59-1∆* and *sec591-1∆* + YEp352-*SEC59* cells were grown in SC or SC-U medium at 30 °C up to mid-log phase. Equal amount of cells was collected, lipid extracted and phospholipid separated by two-dimensional TLC and quantified, as described in the methods section. The data represents the mean ± SD (*P < 0.05 and **P < 0.01) of triplicates from three separate experiments. (**B,D**) GC/MS. Fatty acid analysis in membrane lipid & storage lipids. The wild-type and *sec59-1∆* cells were grown in YPD medium at 30 °C until mid-log phase. Equal amount of cells was collected, lipid extracted, and the individual phospholipids and neutral lipids separated by TLC and re-extracted from the silica gel with chloroform/methanol (2:1, v/v) then subjected to methanolysis using BF3/methanol for the conversion of methyl esters. Fatty acid methyl esters were quantified by gas chromatography mass spectrometry (GC/MS), as described in the methods section. The data represents the mean ± SD (*P < 0.05 and **P < 0.01) of triplicates from three separate experiments. (**C**) Microarray, expression of genes involved in the fatty acid metabolism.

In the *sec59-1∆* cell, palmitoleic acid (C16:1) and oleic acid (C18:1) level were predominantly increased, whereas palmitic acid (C16:0) and C18:0 (Stearic acid) levels were reduced in PC, PE, and PI. In the PS, C16:1, C18:1, and C18:0 were increased but C16:0 was not altered in the sec59*-1*∆ cell when compared to the wild-type (Fig. 7B). In the non-polar lipid, the C16:1 and C18:1 levels were increased whereas the C16:0 and C18:0 were decreased in TAG, DAG, and SE of the *sec59-1∆* compared to the wild-type (Fig. 7D). There was a shift towards the un-saturation of fatty acids in the phospholipids and neutral lipids during the protein glycosylation defect in the *sec59*-1∆ when compared to wild type cell.

The microarray results revealed that the genes involved in fatty acid synthesis, elongation, and the regulatory genes were up-regulated (Fig. 7C). Moreover, 13 genes (*ACS2, FAS1, FAS2, ELO1, FAA1, FAA2, ETR1, EHT1, HFA1, TES1*, and *ACB1*) were up-regulated, whereas only four genes (*OLE1, CEM1, FAA3*, and *FAT3*) were down regulated in the *sec59*-1∆ cell (Fig. 7C).

The shift towards more unsaturated membrane lipids in the *sec59-1*∆ cell would be accompanied

by major rearrangements in lipid classes for maintaining membrane shape and fluidity during the glycosylation defects.

### *SEC59-1* mutation attenuates peroxisome biogenesis

**Figure 8:**
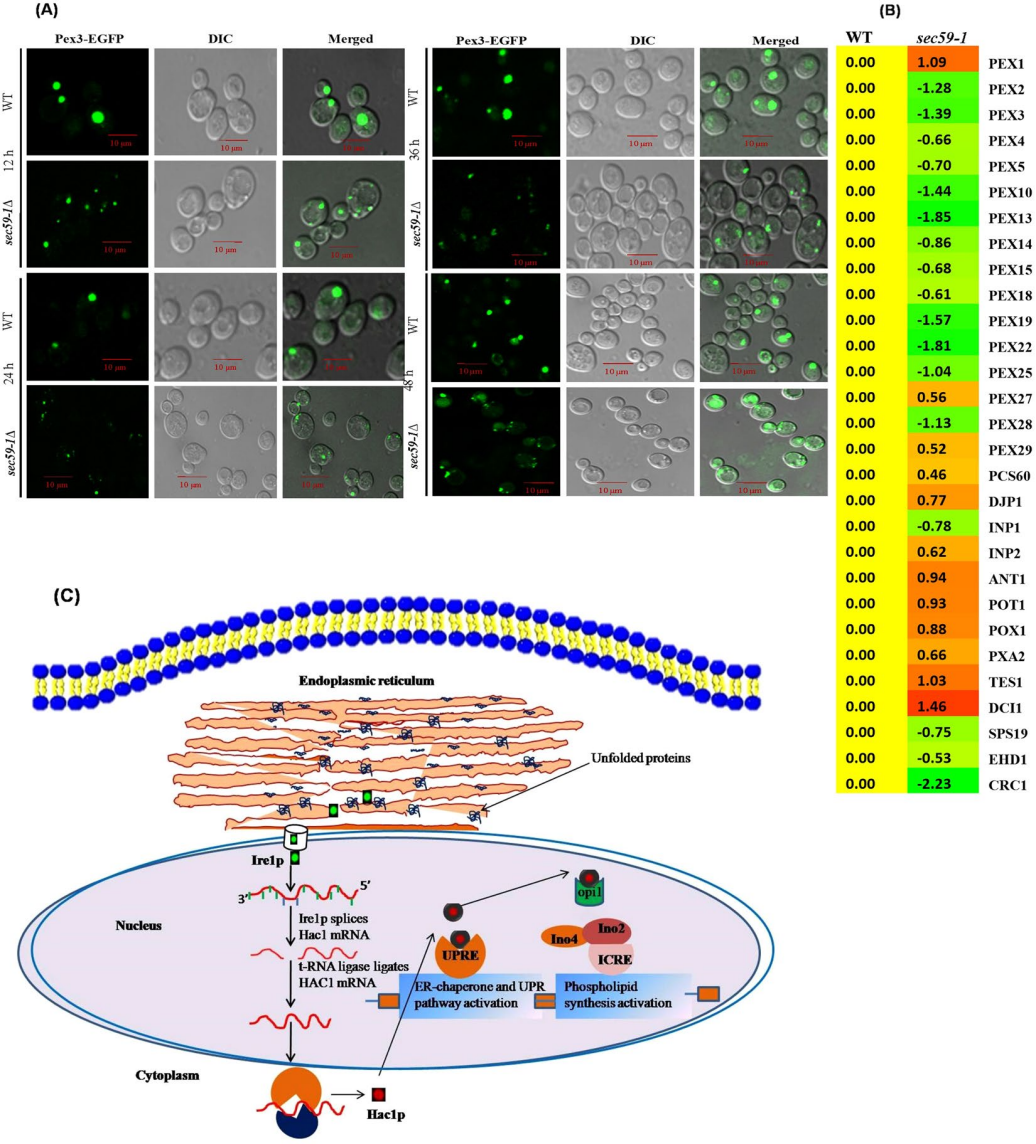
Study of peroxisome biogenesis using the Pex3-EGFP overexpression. (**A**) Wild-type + pIH024-PEX3-EGFP and *sec59-1∆* + pIH024-PEX3-EGFP cells were grown in synthetic complete medium (SC-His) devoid of histidine, harvested in a time-dependent manner (12 h–48 h) at 180 rpm and washed with 1x PBS. Fluorescence imaging was performed on a confocal microscope (LSM 710-Zeiss) equipped with a 100/1.40 oil objective and an AxioCam MRM camera (Zeiss). Bar, 10 μm. (**B**) Microarray, expression of genes involved in the peroxisome biogenesis and beta-oxidation. (**C**) Schematic representation of the mechanism of protein glycosylation defect induced ER stress dysregulates lipid metabolism. During the protein glycosylation defect unfolded proteins accumulated in the ER, inducing the splicing of the transcription factor hac1 through ire1p ribonuclease, thereby inactivating the opi1p negative regulator of lipid synthesis and de-repressing the transcription of phospholipid genes.

In the *sec59-1∆* cell, free fatty acid, neutral lipids (TAG and SE), and LDs were increased, in order to elucidate the impact of *SEC59* defect on peroxisome biogenesis. Peroxisomes plays a crucial role in β-oxidation of fatty acids^26^ and 32 genes (peroxins) have been involved in peroxisome biogenesis^27,28^. The Pex3p is a peroxisomal membrane protein and highly conserved from yeast to mammalian system that regulates peroxisome dynamics and peroxisome biogenesis by interacting with Pex19p^29^. The microarray results showed that more than 12 major “peroxin” genes (*PEX2, PEX3, PEX4, PEX5, PEX19, PEX10, PEX13, PEX14, PEX15, PEX18, PEX22*, and *PEX28*) were down regulated whereas only *PEX1* was up-regulated, and rest of the peroxin genes (*PEX6, PEX7, PEX8, PEX11, PEX12, PEX17, PEX21, PEX27*, and *PEX29*) were not significantly altered in the *sec59-1∆* cell (Fig. 8B,C). The peroxisome biogenesis defect was further confirmed using PEX3-EGFP (enhanced green fluorescence protein) overexpression. The biogenesis and maintenance of peroxisomes from ER-derived membrane components required Pex3p, which is independent of the oleic acid induced peroxisome biogenesis and proliferation^30,31^. In the *sec59-1Δ* cell, discrete punctate of Pex3-EGFP was reduced in the peroxisomal structures whereas the large and puncta peroxisome structure was observed in wild-type (Fig. 8A). The Pex3-EGFP fluorescence was also drastically reduced in the *sec59-1∆* strain. In order to determine if the peroxisome proliferation defect was eventually recovered in the *sec59-1∆* cells we looked at time lapse experiments which showed that the reduced peroxisome size and discrete structure in the *sec59-1∆* cell even at 48 h. The peroxisomes appeared as dispersed and fragmented structures with various sizes at 24 h and 36 h resulting in a dramatic loss of peroxisomes structure and subsequently, PEX3-EGFP moved to vacuoles at 48 h (Fig. 8A). From these results, we can conclude that defect in dolichol kinase plays a major role in peroxisome biogenesis and maintenance.

## Discussion

The present study reports the link between protein glycosylation and lipid metabolism, and its impact on the ER protein quality control, and peroxisome biogenesis in *S. cerevisiae*. The growth of the *Sec59-1∆* strain was significantly reduced even in permissible temperature compared to wild-type (Figs 1D and S1). During the mutation of Sec59-1, the N-glycosylation of vacuolar carboxypeptidase-Y (CPY) was severely affected, and simultaneously UPR was constitutively induced as an adaptive response to protect the cells from stress. The ER stress was increased as noted by increased expression of Kar2p which could be due to a protein glycosylation defect in the *sec59-1∆* strain (Fig. 2A,B). In the *sec59-1∆* strain, the intra-cellular membrane aggregation and fragmented membrane vesicles were increased (Fig. 1E). This pleiotropic phenotype could be due to a protein glycosylation defect and a similar phenotype has been reported in previous study^32,33^. The microarray results revealed that 14 genes involved in cell membrane synthesis, morphology maintenance, and cell wall glycan synthesis were up-regulated in the *sec59-1Δ* cell. Among them the *SCS3* (involved in ER membrane proliferation) was up-regulated whereas *RTN1* (involved in ER membrane integrity and morphogenesis) was down regulated in the *sec59-1∆*strain (Fig. 1F). These results could be attributed to increased ER membrane proliferation, and intercellular membrane vesiculation in the *sec59-1Δ* strain. The reduced membrane integrity and morphogenesis was depicted by an increases of fluorescence with DiOC6 implying the alteration of plasma membrane in the *sec59-1*Δ cell (Fig. 2E and S1B). To protect the cells from ER stress, the misfolded or unfolded proteins activate UPR as an adaptive response increasing the ER chaperone which could promote refolding of the protein. During the mutation of *Sec59-1*, the ER chaperones and UPR regulatory genes were increased in order to protect the cells from ER stress (Fig. 2D). Further, there was increased expression of genes involved in the ERAD pathway (*DOA10, DFM1, UBX4, UFD1, HRD3, USA1*, and *DER1*) which can eliminate the misfolded or unfolded proteins from the ER for the cell survival under ER stress.

Apart from the protein synthesis and quality control maintenance, ER plays a major role in synthesis of membrane phospholipids and sterol. Lipid homeostasis is regulated by ER sensors through the transcriptional and post-transcriptional mechanisms^13^. In *S. cerevisiae*, membrane phospholipid and storage lipid classes recruit DAG from the precursor phosphatidic acid (PA). During the exponential growth phase, DAG is predominantly channeled to phospholipid synthesis, and required for membrane formation, cell viability, and growth, whereas the phospholipid biosynthesis is reduced during stationary phase^34^. In contrast, the levels of all major phospholipids (PC, PE, PI, and PS) were significantly increased from mid-log phase to stationary phase in the *sec59-1∆* cell compared to the wild-type (Fig. 3A,B). Also, the microarray results revealed that increased expression of phospholipid genes due to down regulation of negative regulator (*OPI1)* of phospholipid synthesis gene (Fig. 3C). This result supports our previous studies, which suggest that increased phospholipid level in protein glycosylation defect and ER stress phenotype strain^32^. The plasma membrane flippase and permease (*GIT1, DNF1*, and *DNF2*) were up-regulated in the *sec59-1∆* cell implying that phospholipid flippases provide the substrates for the CDP-choline and ethanolamine pathway. The derepression of PE-methylation and Kennedy pathway genes could account for the increased phospholipid synthesis in the *sec59-1∆* cell. The increased phospholipid synthesis could be a protective response by increasing the hydrophobic surface of the ER, thereby sequestering misfolded proteins in response to ER stress^35^.

In the *sec59-1∆* cell, the increased level of PI by the upregulation *INO1* and *PIS1*, and increased expression of genes involved in dephosphorylation of phosphatidyl inositol phosphates (*INP52, INP53, SAC1*) (Fig. 3A–C). PI is the most predominant lipid in the ER, which protects the cells from stress by increased expression of *INO1*.

Apart from the phospholipid, fatty acids are playing a crucial role in cellular stress response and have been reported in both *S*. *cerevisiae* and mammals^36^. In the *sec59-1*∆ cell, the C16:1 and C18:1 level were predominantly increased in PC, PE, PI, and PS, whereas C16:0 and C18:0 were reduced, except PS where it was not altered. Apart from the ER, phospholipids are synthesized in mitochondria, vacuoles, and Golgi apparatus. During the Sec59-1 mutation, PE, and CL level were significantly increased at mid-log phase (Fig. S1D), and the expression of genes involved in PE and CL synthesis (*EPT1, ALE1, FMP30*, and *CRD1*) were upregulated. This could be attributed to the increased levels of PE and CL observed in the *sec59-1Δ* cell (Fig. 3A–C). Furthermore, the major mitochondrial fusion and fission gene expression were also affected. The mitochondrial heat shock proteins are associated with mitochondrial unfolded response (mito-UPR), and the genes expressions were up-regulated. These cellular complications induced the apoptosis genes in the *sec59-1Δ* cell (Fig. S1F). These results depict the dysfunction of the mitochondrial genes in the *sec59-1Δ* cell.

In *S. cerevisiae*, during the stationary phase, the DAG and fatty acids are predominantly channeled to TAG synthesis and stored as LD in the cytosol. In the *sec59-1∆* cell, TAG and SE levels were drastically increased. Also, the sterol, 1, 2-DAG, 1, 3-DAG, and free fatty acids were increased when compared to the wild-type (Fig. 6A–C). The genes involved in the sterol metabolism and its regulatory genes were increased (Fig. 5B,C). The accumulation of sterol in the *sec59-1∆* cell could be due to reduced transport from the ER to the plasma membrane by the down-regulation of *ARV1* and increased transport of sterol from the plasma membrane to the ER by the derepression of *YSP1, YSP2*, and *SIP3* (Fig. 5B). The LD number and size were increased as measured by the increased intensity of Nile red fluorescence when compared to the wild-type (Fig. 6C). Also, the time lapse study revealed drastic increases of TAG, SE, and LDs in the *sec59-1∆* strain (Fig. 4A,B) which is supported by increased expression of *LRO1* and *ARE2*, and down-regulation of major TAG and SE lipases (*TGL3, TGL5*, and *YEH2* and *TGL1)* (Fig. 5A). Our previous study shows that LD and neutral lipids were reduced in protein glycosylation defect phenotype (*Cax4∆*) due to specific N-glycosylation defect. The *Sec59-1∆* phenotype results controversy to *Cax4∆* strain may due to defect in multiple glycosylation defects including, N-glycosylation, O-glycosylation, and GPI anchor synthesis. *DPP1* (Zn dependent phosphatidate phosphatase) was up-regulated in the *sec59-1∆* could be account for reduced transport of zinc by the down regulation of vacuolar membrane zinc transporter *(ZRT*3). These results strongly revealed the vacuolar lipid phosphatase plays a role for the supply of substrate (DAG) to TAG synthesis when the Zn transport get affected in the *sec59-1∆* cell (Fig. 5A).

The *Sec59-1* cells exhibit increases the unsaturated fatty acids, especially C16:1, and C18:1 in neutral lipids and phospholipids. The only gene that has been reported to involved in the synthesis of mono-unsaturated fatty acids is *OLE1* (delta 9-fatty acid desaturase)^37^, but its expression was significantly reduced in the *sec59-1∆* cell. This result suggests that there may be other desaturases in *S. cerevisiae* that are yet to be identified under the stress.

LD is a ubiquitous intracellular organelle, a storage depot that provides energy during starvation and involved in membrane lipid synthesis. LD biogenesis and growth are uniquely dependent on neutral lipid synthesis. LD’s are often closely associated with various organelles, including peroxisomes, mitochondria, vacuole, and endoplasmic reticulum (ER). ER plays a potential role in peroxisome biogenesis and triggers the targeting signal of peroxisomal membrane proteins (PMPs) to peroxisomes. The increased lipolysis of TAG and SE increases free fatty acids, which undergo β-oxidation in the peroxisome. In *sec59-1∆* cell, the TAG, SE, LD’s, and FFA were increased and subsequently, the genes involved in β-oxidation (*POX1, POT1, IDP2, ECI1, TES1*, and *DCI1*) were also up-regulated (Fig. 8B).

The microarray result revealed that 12 genes involved in peroxisome biosynthesis were down regulated whereas the *PEX1* was up-regulated, and the rest of the genes were not altered (Fig. 8B). Furthermore, the peroxisome biogenesis defect was confirmed by the reduced PEX3-EGFP fluorescence in the *sec59-1∆* when compared to the wild cell. These results suggest that dolichol kinase dependent protein glycosylation directly or indirectly plays a role in peroxisome biogenesis and β-oxidation. Finally, we can conclude that impaired growth of the Sec59-1Δ cells is associated with constitutive unfolded protein response, ER stress, and dysregulation of lipid metabolism accounting for the pleiotropic phenotypes. Thus, the *S. cerevisiae* dolichol kinase controls membrane biogenesis by coordinating lipid homeostasis with protein quality control.

## Materials and Methods

The yeast strains used in this study (WT-RSY252-MATa; his4-619; ura3-52 and RSY27 MATa; his4-493; ura3–52; suc-432; *sec59-1∆* KanMAX4) were gifted by Prof. Randy Schekman (Howard Hughes Medical Institute and Department of Molecular and Cell Biology, University of California, USA) The plasmid YEp352-*SEC59* and its control vector were gifted by Prof. Markus Aebi (Institute of microbiology, ETHZ, Zurich, Switzerland). The plasmid pIH024-PEX3-EGFP was a generous gift from Prof. Dr. Harald W. Platta (Institute of Biochemistry and Pathology, Department of Biochemistry, Germany).The anti-kar2 antibody was a gift from Prof. Jeffrey L. Brodsky (Department of Cell Biology, University of Pittsburgh, PA and USA), and anti-CPY antibody was gifted by Prof. Neta Dean (Department of Biochemistry and Cell Biology, Stony Brook University, NY and USA). Yeast nitrogenous base (YNB), yeast extract and peptone were obtained from Difco, the nitrocellulose membrane was purchased from Millipore (India Pvt. Ltd), and thin layer chromatography plates were obtained from Merck (India). Phospholipids standard, ampicillin, kanamycin, synthetic complete media (SC), solvents and all other reagents were procured from Avanti polar lipids and Sigma, St. Louis, USA unless specifically mentioned.

### Growth conditions

Yeast cells were grown in YPD (1% yeast extract, 2% peptone, and 2% dextrose) medium (pH 7.0) with aeration at 30 °C until mid-log phase. For lipid studies, cells were grown in 5 ml of YPD medium, and cell mass was measured at A_600 nm._ The equal number of cells was harvested at frequent time intervals for lipid extraction and confocal fluorescent microscope studies. The plasmid-bearing cells were grown in synthetic complete (SC) media with appropriate amino acids and devoid of uracil.

### Growth study

The wild-type, wild + YEp352, *sec59-1∆*, and *sec59-1∆* + YEp352-*SEC59* cells were grown in YPD and SC or SC-U medium at 30 °C up to mid-log phase. Cell mass was measured at A_600 nm,_ and equal mass of cells were collected and serially diluted from 10^−1^ to 10^−5^ dilution and three μl of cells were spotted on (YPD and SC or SC-U) appropriate agar plates, and then incubated the plates for 48 h at 30 °C.

### Lipid extraction and separation

Lipids were extracted from the yeast cells by the method of Bligh and Dyer^38^. Briefly, to the cell pellet 400 µl of methanol and 200 µl of chloroform were added and vortexed. Further, 400 µl of acidified water (2% orthophosphoric acid) was added and vigorously vortexed. The lipids were extracted with Chloroform and subjected to two-dimensional thin layer chromatography (TLC) with Silica Gel 60F254 TLC plates^39^. The phospholipid were separated using the following solvents: for first dimension chloroform/methanol/ammonia (65:25:5, v/v) and for the second dimension, chloroform/methanol/acetone/acetic acid/water (50:10:20:15:5, v/v). Individual lipids were located by comparing the *Rf* value of the unknown with the *Rf* value of the standard. Spots corresponding to individual phospholipids were scraped off and quantified^40^. For analyzing the neutral lipids (TAG, and SE), the lipids were separated using petroleum ether/diethyl ether/acetic acid (70:30:1; v/v) with triolein and cholesteryl oleate as standards, and lipids were quantified with slight modifications^41^. Briefly, the plates were dipped in methanolic MnCl_2_ solution (0.63 g MnCl_2_·4H_2_O, 60 ml of water, 60 ml of methanol, and 4 ml of concentrated sulfuric acid), dried, and heated at 120 °C for 15 min. Densitometric scanning was performed at 500 nm with a CAMAG TLC Scanner.

### Fatty acids analysis

The phospholipids and neutral lipids separated in the TLC were extracted using chloroform/methanol (2:1, v/v), and subjected to methanolysis form conversion to methyl esters using BF3/methanol^42^. Fatty acid methyl esters (FAME) were separated and quantified by gas chromatography mass-spectrometry (GC/MS). The heptadecanoic acid methyl ester (C17:0) was used as an internal standard for fatty acid quantification.

### Nile Red staining

The wild-type, sec59*-1*∆, and *sec59-1*∆ + YEp352-*SEC59* cells were grown in SC or SC-U medium at 37 °C and 180 rpm. The cells were harvested at mid-log phase and washed with 1x PBS and stained with lipophilic dye Nile Red. Fluorescence imaging was performed on the confocal laser scanning microscope (LSM 710-Zeiss) equipped with a 100/1.40 oil objective and an AxioCam MRM camera.

### DiOC6 Staining (3,3′dihexyloxacarbocyanine Iodide)

The wild-type, *sec59-1*∆ and *sec59-1*∆ + YEp352-*SEC59* cells were grown in SC or SC-U medium at 180 rpm. Cells were harvested at mid-log phase and washed with 1x PBS then cells were stained with one μl of lipophilic dye DiOC6 (1 mg/ml ethanol). Fluorescence imaging was performed immediately after staining on the confocal laser scanning microscope (LSM 710-Zeiss) equipped with a 100/1.40 oil objective and an AxioCam MRM camera.

### Yeast transformation

The yeast plasmid YEp352 + *SEC59*, pRS314 + UPRE-GFP, and pIH024 + *PEX3*-EGFP was transformed into wild-type and *sec59-1*∆ cells by the lithium acetate transformation method^43^, and the transformants were selected on plates containing SC-U, SC-Try, SC-Leu (synthetic complete medium devoid of uracil, tryptophan and leucine) minimal medium (0.67% YNB, 2% dextrose and complete supplement mixture lacking respective amino acids).

### Fluorescence measurement of UPRE-GFP

The wild-type + pRS314-UPRE-GFP and *sec59-1*∆ + pRS314-UPRE-GFP plasmid bearing cells were grown in SC-Try medium at 180 rpm in 30 °C. The cells were collected every 12 h intervals (12 h to 48 h), then cell mass was measured at A_600 nm_ and equal amount (1.0 OD) of cells were resuspended with 1 ml 1x PBS buffer (8 g/L NaCl, 1.44 g/L Na_2_HPO_4_, 0.24 g/L KH_2_PO_4_, 0.2 g/L KCl). The GFP intensity was measured on an F-4500 Fluorescence Spectro-photometer (Hitachi). The measurements were carried out at 488 nm (excitation) and 510 nm for the emission wavelength.

### Microarray analysis

For the preparation of RNA, the wild-type and *sec59-1*∆ cells (0.1 OD_600_) were grown until 48 h in YPD medium and harvested by centrifugation. Cell pellets were shock frozen in liquid nitrogen and stored at −70 °C until further use. Total RNA was isolated using an RNeasy Kit (Qiagen) including DNase I treatment according to the manufacturer’s instructions. Yeast lysate was prepared by mechanical disruption of cells using a bead mill. The integrity of RNA was tested by agarose gel electrophoresis. The RNA concentration was estimated by the A_260 nm_ and the purity determined A_260 nm_ A_280 nm_ ratio. For expression profiling, 1 μg of total RNA was linearly amplified and biotinylated using the One-Cycle Target Labeling Kit (Affymetrix, Genotypic, Bangalore, India) according to the manufacturer’s instructions. The labeled and fragmented cRNA (15 μg) were hybridized to Affymetrix Yeast 8.0 Gene Chip®arrays (Affymetrix). After hybridization, the arrays were washed and stained in a Fluidics Station 450 (Affymetrix) with the recommended washing procedure. Biotinylated cRNA bound to target molecules was detected with streptavidin-coupled phycoerithrin, biotinylated anti-streptavidin IgG antibodies and again streptavidin-coupled phycoerithrin according to the manufacturer’s protocol. Arrays were scanned using the GCS3000 Gene Chip scanner (Affymetrix) and GCOS 1.4 software. Scanned images were subjected to visual inspection to test for hybridization artifacts and proper grid alignment, and analyzed with Microarray Suite 5.0 (Affymetrix) to gene ratereport files for quality control. Statistical data analysis was performed using the bioconductor packages “affy” and “limma.” Initially, the expression data from all arrays were normalized with RMA to yield log2-transformed signal values. The assay was performed in two independent experiments with two and four samples, respectively. A linear model was generated including the factors “batch” and “strain” to correct for this effect. The signal values were then averaged for the individual subgroups and differences in the expression level were calculated as x-fold change. Differences between *sec59-1*∆ and the wild-type strains were extracted and analyzed with the moderated T-test (empirical Bayes method). The transcripts with at least value 0.6 changes in the expression level and a p-value of <0.05 were regarded as differentially expressed. Data extraction from Images was done using Feature Extraction software v 10.7 of Agilent. The extracted data were analysed using Gene Spring GX version 11 software from Agilent. Normalization of the data was done in Gene Spring GX using the 75^th^ percentile shift and normalization to specific samples.

### Western blotting

Yeast cells were grown in YPD or SC or SC-U media, harvested every 12 h intervals (12 h–48 h) and the cells were centrifuged at 6,000 rpm for 5 min. The cells were washed and suspended in lysis buffer containing 50 mM Tris HCL, pH 7.5, 150 mM NaCl, 5 mM EDTA, and 1 mM phenylmethylsulfonyl fluoride (PMSF). Cells were lysed using glass beads and the unbroken cells were removed by centrifugation at 10,000 rpm for 15 min at 4 °C and the supernatant was collected and protein determined (Bradford 1976).

The protein (50 µg) was subjected to 8% SDS-PAGE for Kar2 and Cpy. To visualize the protein bands in the gel, SDS-PAGE gels were stained by coomassie brilliant blue staining. For immunoblot analyses, proteins were transferred to nitrocellulose membrane at 120 V for 2 h and incubated with a 1:2,500 dilution of anti-Kar2p or anti-Cpy1p for 2 h, followed by a 1:2,500 dilution of ALP-conjugated goat anti–rabbit IgG for 1 h. The membrane was washed again and detected with BCIP/NBT substrate.

### Statistics

Data were analysed using the programs of SPSS 10.0 (Statistical Package for the Social Science for Windows 11.5). All the values reported in the study were the mean of three replicates. Statistical analysis was carried out by analysis of variance (ANOVA) test. Significant differences among mean was determined at *p < 0.05, **p < 0.01, and ***p < 0.001. The microarray gene expression data were analyzed and differential expression values are provided as log base 2. The value at least 0.6 is statistically significant (*p < 0.05).

### Accession codes

Microarray and sample annotation data were deposited in NCBI’s Gene Expression Omnibus (GEO) data repository under accession no. GSE70809.

## Supplementary information

Data set 1

Dataset 2

Dataset 3

Data set4
