# Supplementary material for: Crosstalk between protein N-glycosylation and lipid metabolism in Saccharomyces cerevisiae
Source: Sci Rep. 2019 Oct 9;9:14485. doi: 10.1038/s41598-019-51054-7 (PMC6785544; doi:10.1038/s41598-019-51054-7)
Supplement: Supplementary file 1 — Data set 1 [file 41598_2019_51054_MOESM1_ESM.docx]

Crosstalk between protein N-glycosylation and lipid metabolism in *Saccharomyces cerevisiae*

Antonisamy William James^1^, Chidambaram Ravi^1^, Malathi Srinivasan^2^ and Vasanthi Nachiappan*^1^

^1^Biomembrane Lab, Department of Biochemistry, School of Life Sciences, Bharathidasan University, Tiruchirappalli, 620 024, Tamilnadu, India.

^2^Department of Lipid Science, CSIR-Central Food Technological Research Institute (CSIR-CFTRI), Mysore, 570020, India.

*Correspondence and requests for materials should be addressed to Vasanthi Nachiappan (email: [vasanthibch@gmail.com](mailto:vasanthibch@gmail.com) and[vasantinr@gmail.com](mailto:vasantinr@gmail.com))

Supplementary figure S1. Cell growth rate Membrane defective cells


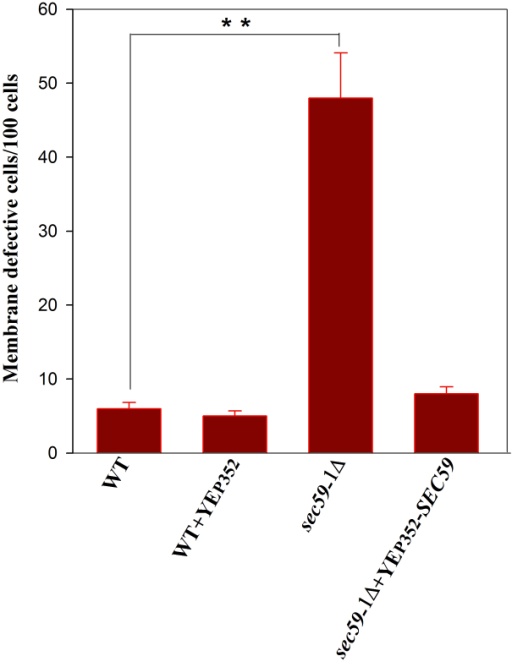
A B


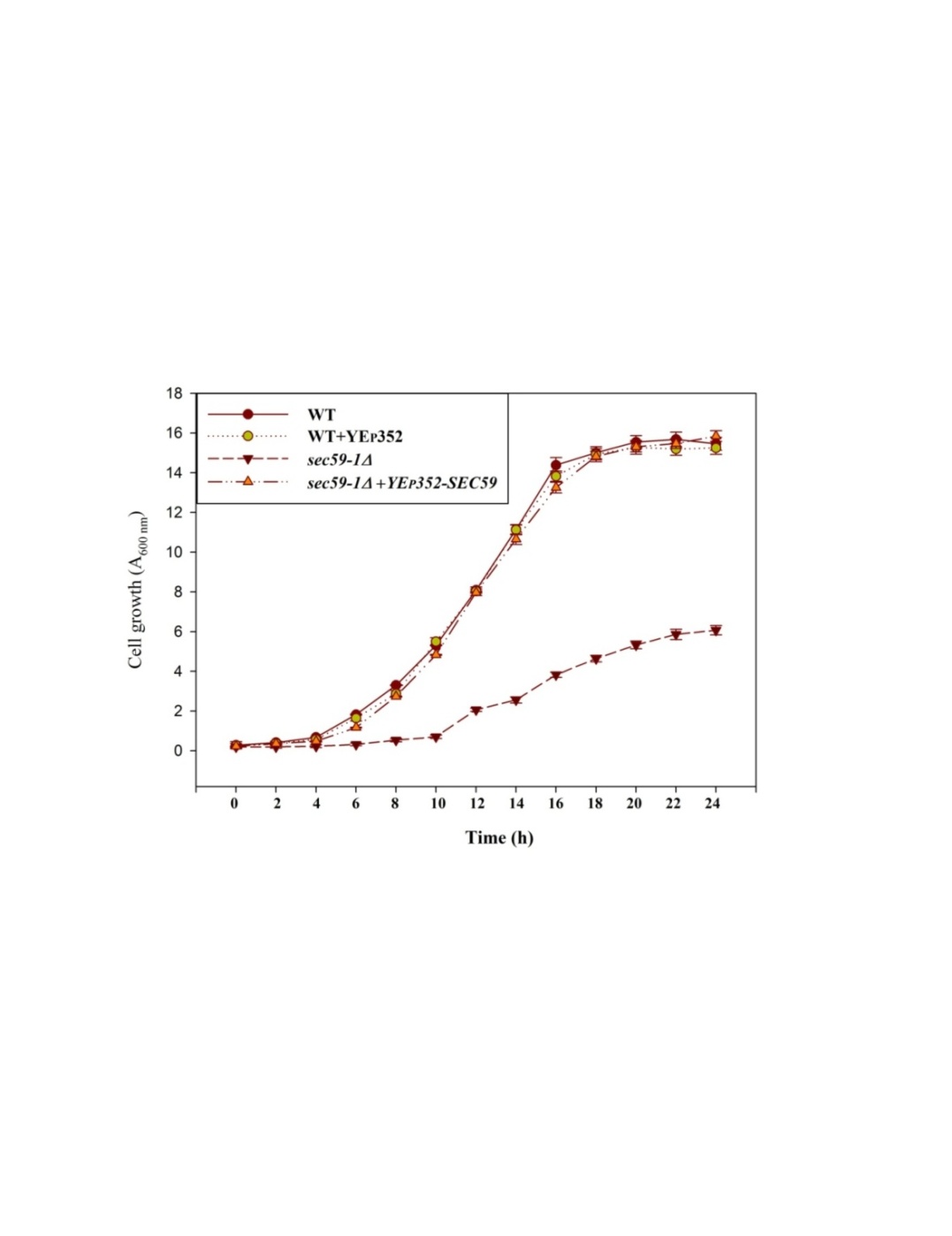


C D


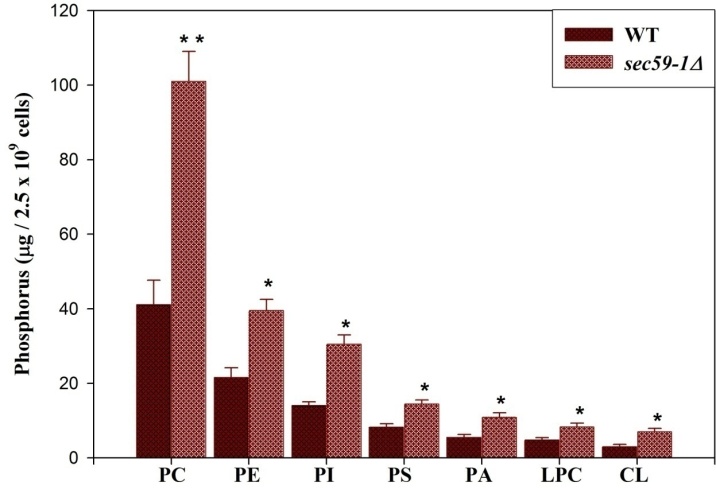

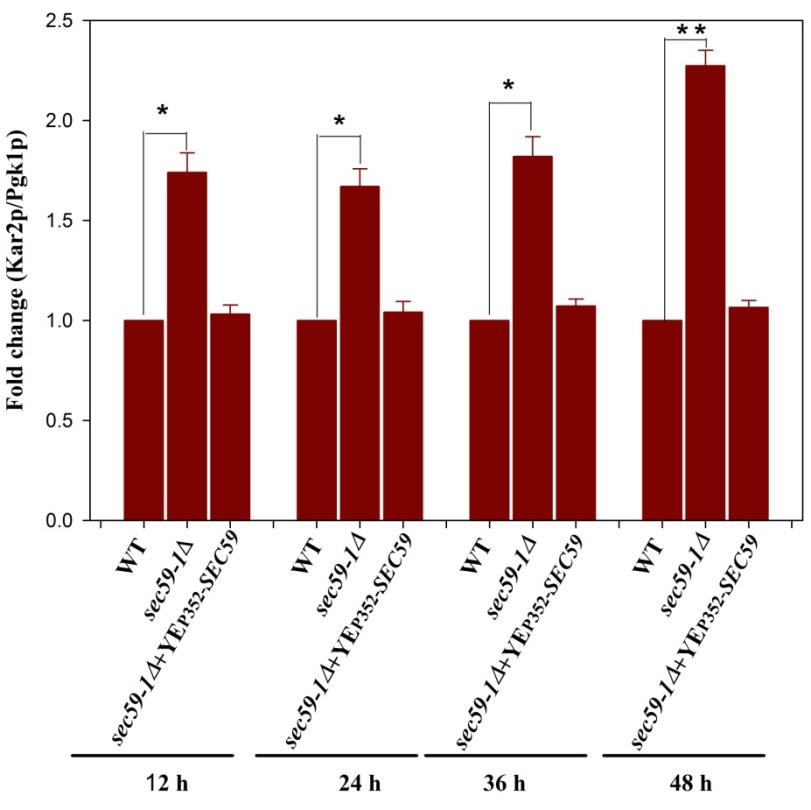


A. Cell growth

B. Membrane defective cells

C. Kar2 protein expression quantification. D. Phospholipid quantification

Supplementary figure.S2

| **WT** | ***Sec59-1Δ*** |  |
| --- | --- | --- |
| **0.00** | **-0.53** | **TAM41** |
| **0.00** | **-0.81** | **GEP4** |
| **0.00** | **-1.20** | **GEP5** |
| **0.00** | **0.50** | **CRD1** |
| **0.00** | **-0.71** | **CLD1** |
| **0.00** | **1.08** | **RTN1** |
| **0.00** | **-1.18** | **UPS1** |
| **0.00** | **0.96** | **FMP30** |
| **0.00** | **-1.14** | **GEM1** |
| **0.00** | **0.68** | **MMM1** |
| **0.00** | **1.43** | **MDM10** |
| **0.00** | **0.88** | **MDM12** |
| **0.00** | **-0.51** | **MDM30** |
| **0.00** | **-0.62** | **MDM34** |
| **0.00** | **-1.17** | **MDM32** |
| **0.00** | **0.85** | **CYM1** |
| **0.00** | **-1.01** | **FIS1** |
| **0.00** | **0.47** | **UGO1** |
| **0.00** | **-0.51** | **MDM30** |
| **0.00** | **-1.20** | **FZO1** |

(B)

WT ***sec59-1∆***

| **0.00** | **0.84** | **AFG1** |
| --- | --- | --- |
| **0.00** | **1.13** | **HSP10** |
| **0.00** | **1.15** | **HSP104** |
| **0.00** | **1.73** | **HSP78** |
| **0.00** | **-1.18** | **AIM38** |
| **0.00** | **2.06** | **YSP2** |
| **0.00** | **1.26** | **LOT6** |
| **0.00** | **2.32** | **AIM14** |
| **0.00** | **-0.86** | **RSM23** |

C.


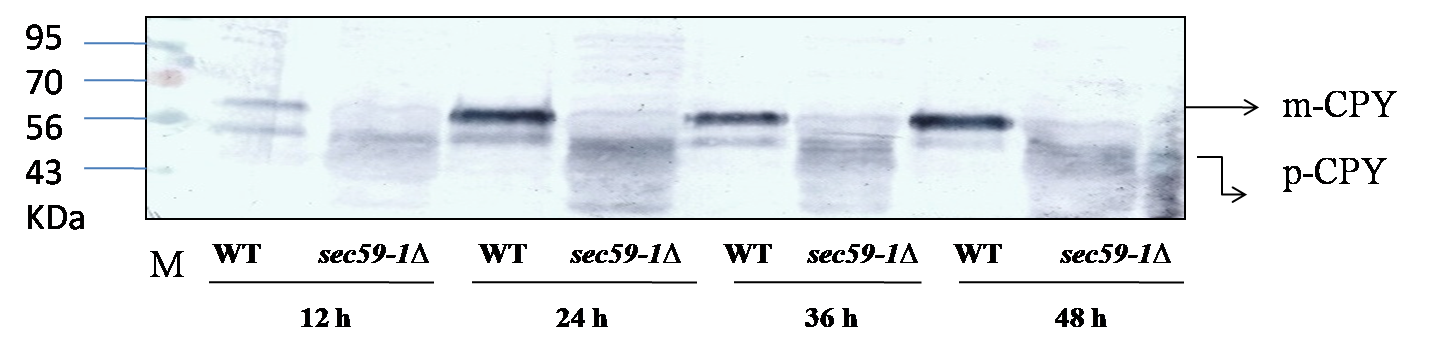


D.


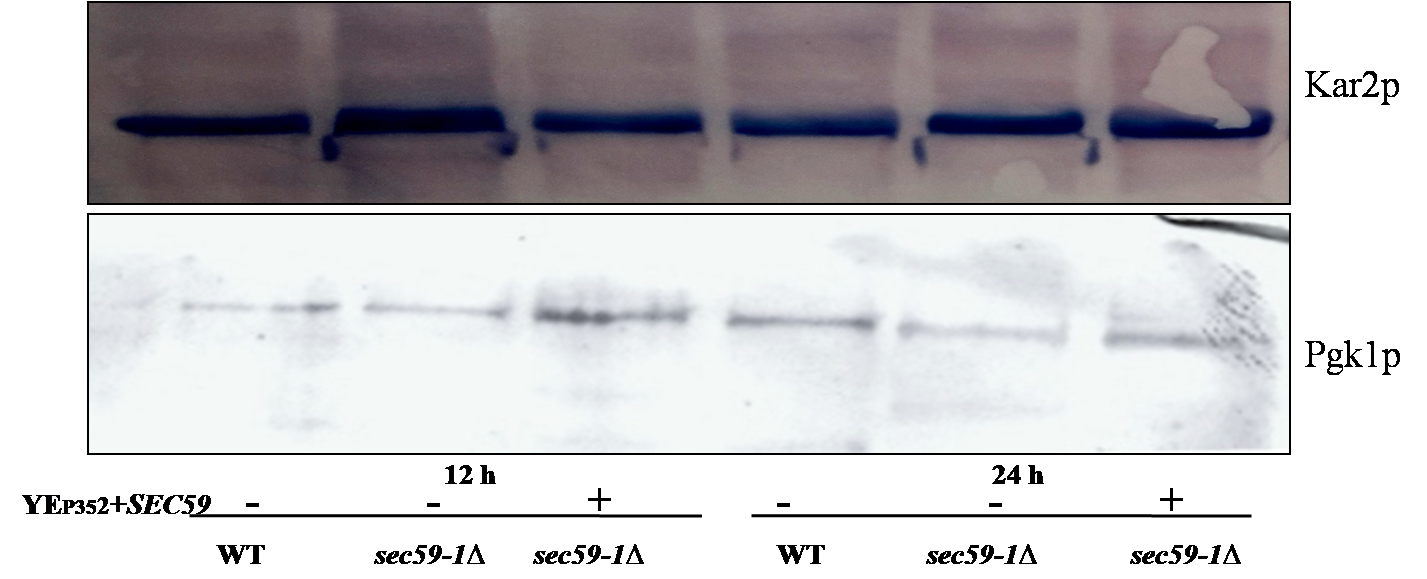


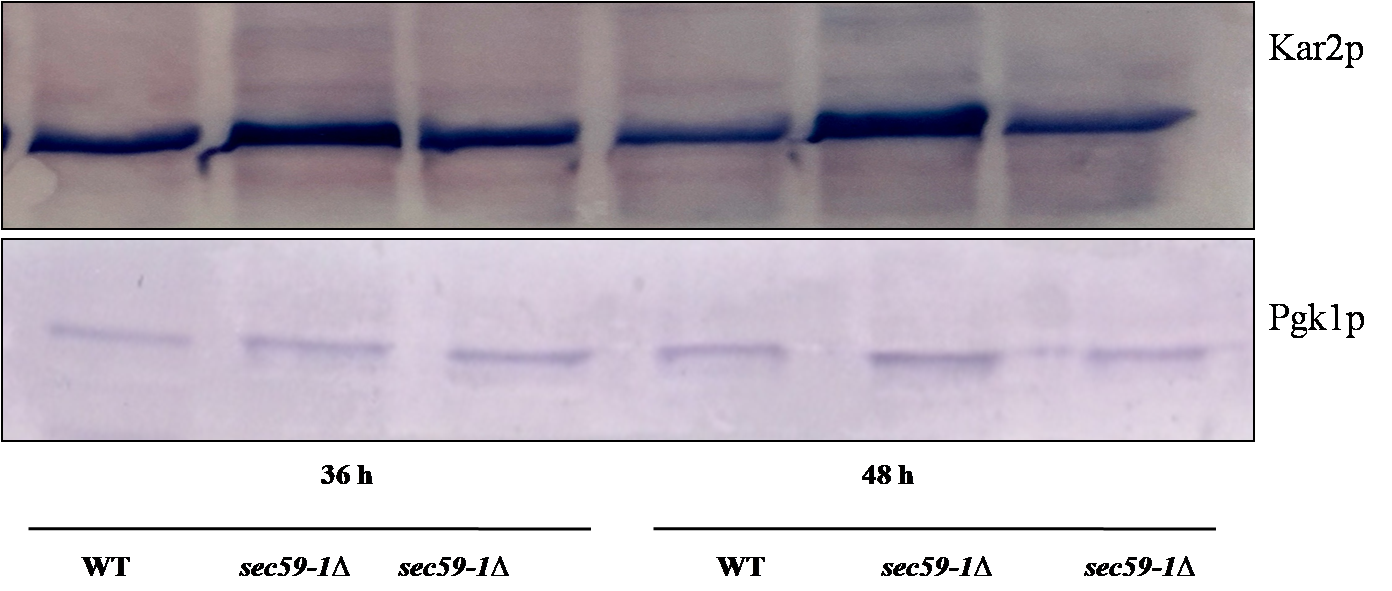


A. The expression of genes involved in the mitochondria lipid synthesis and morphology genes.

B. The expression of genes involved in apoptosis and protein folding.

C. Expression of CPY in wild-type and sec59-1∆ cells.

D. Expression of kar2
